# *Candida parapsilosis* Virulence and Antifungal Resistance Mechanisms: A Comprehensive Review of Key Determinants

**DOI:** 10.3390/jof9010080

**Published:** 2023-01-05

**Authors:** Joana Branco, Isabel M. Miranda, Acácio G. Rodrigues

**Affiliations:** 1 Division of Microbiology, Department of Pathology, Faculty of Medicine, University of Porto, 4200-319 Porto, Portugal; 2 Center for Health Technology and Services Research—CINTESIS@RISE, Faculty of Medicine, University of Porto, 4200-450 Porto, Portugal; 3 Cardiovascular Research & Development Centre—UnIC@RISE, Faculty of Medicine, University of Porto, 4200-450 Porto, Portugal

**Keywords:** fungal infections, *Candida* spp., *Candida parapsilosis*, virulence attributes, polyenes, echinocandins, azoles, antifungal resistance, biofilm formation, healthcare-related infections

## Abstract

Candida parapsilosis is the second most common Candida species isolated in Asia, Southern Europe, and Latin America and is often involved in invasive infections that seriously impact human health. This pathogen is part of the psilosis complex, which also includes Candida orthopsilosis and Candida metapsilosis. C. parapsilosis infections are particularly prevalent among neonates with low birth weights, individuals who are immunocompromised, and patients who require prolonged use of a central venous catheter or other indwelling devices, whose surfaces C. parapsilosis exhibits an enhanced capacity to adhere to and form biofilms. Despite this well-acknowledged prevalence, the biology of C. parapsilosis has not been as extensively explored as that of Candida albicans. In this paper, we describe the molecular mechanistic pathways of virulence in C. parapsilosis and show how they differ from those of C. albicans. We also describe the mode of action of antifungal drugs used for the treatment of Candida infections, namely, polyenes, echinocandins, and azoles, as well as the resistance mechanisms developed by C. parapsilosis to overcome them. Finally, we stress the importance of the ongoing search for species-specific features that may aid the development of effective control strategies and thus reduce the burden on patients and healthcare costs.

## 1. *Candida* and Human Disease

Fungi can cause a diversity of health disorders in humans, ranging from allergic syndromes and mucocutaneous infections to invasive diseases that seriously threaten life. It is estimated that fungal diseases annually affect over a billion people and cause 1.5 million deaths worldwide [1]. Invasive fungal infections caused by *Candida* species are widely associated with high rates of severe illness and may be responsible for as many as 30% of all deaths from fungal disease. In the United States, the health cost attributable to prolonged hospitalizations resulting from candidaemia is estimated at USD 46,684 per patient [2].

Candidosis is a broad term that refers to cutaneous, mucosal, and deep-seated organ infections caused by opportunistic pathogens of the *Candida* genus [3]. *Candida* spp. are commensal yeasts commonly found in the human gastrointestinal tract, mucous membranes, and skin. Disruption of the gastrointestinal and cutaneous barriers following shock, localized infections, or the replacement of an intravascular catheter can all promote invasive candidosis, which is widely recognized as a major cause of morbidity and mortality. The patient populations most at risk are the elderly, premature newborns, and those with compromised immune systems due to HIV, chemotherapy, or transplant-necessitated immunosuppression therapy [4]. Invasive candidosis is a disorder that can potentially affect any organ. Each distinct *Candida* species exhibits its own unique characteristics in terms of its invasive potential, virulence, and antifungal susceptibility pattern [3].

The distribution of *Candida* species varies geographically, with notable differences between hospital centers. The underlying condition of the patient and whether they have experienced previous antifungal therapy both have an effect on the distribution and frequency of *Candida* spp. [5]. While *C. albicans* is the most common pathogen associated with nosocomial invasive candidosis worldwide, an increasing number of infections by non-albicans *Candida* species (NACs) have also been reported in recent years, including *Candida glabrata*, *Candida parapsilosis*, *Candida tropicalis*, *Candida krusei,* and *Candida auris*, among others [6]. Of these, *C. glabrata* predominates in Northern European countries and in the United States, but *C. parapsilosis* and/or *C. tropicalis* are more prevalent in India, Pakistan, Latin America, and Mediterranean countries [3].

## 2. *Candida parapsilosis*

Since its discovery in 1928, *C. parapsilosis* has undergone several changes in phylogenetic classification. Initially isolated from the stool of a patient with diarrhea in Puerto Rico, the species was first classified as *Monilia parapsilosis* (i.e., a species of the *Monilia* genus, incapable of fermenting maltose) to distinguish it from *Monilia psilosis*, which is today known as *C. albicans* [7]. In 1932, it was renamed *Candida parapsilosis*. In 2005, Tavanti et al. [8] confirmed, through multilocus sequence typing, the existence of a *C. parapsilosis* complex comprising three distinct species: *Candida parapsilosis sensu stricto*, *Candida orthopsilosis,* and *Candida metapsilosis*. In this paper, we focus on *Candida parapsilosis*.

*C. parapsilosis* is widely distributed in nature and is often isolated from a variety of non-human sources, such as domestic animals, insects, soil, and marine environments [9]. This yeast successfully colonizes the human skin and mucosal membranes as a commensal microorganism, wherein the hands of healthcare professionals are recognized as a major vector for *C. parapsilosis* nosocomial acquisition [10,11,12]. In addition, the selective ability of *C. parapsilosis* to grow in hyperalimentation solutions promotes the infection risk by this pathogen [13]. *C. parapsilosis* represents a high risk for immunocompromised individuals, such as HIV sufferers and surgical patients, particularly those subjected to gastrointestinal track surgery. Additionally, patients requiring prolonged use of a central venous catheter or other indwelling devices are at high risk, due to the innate ability of *C. parapsilosis* to adhere to prosthetic surfaces and implanted medical devices. In such cases, biofilm formation typically begins soon after attachment. When the structure is mature, it greatly decreases the ability of antifungals to reach cells, with potentially life-threatening consequences in the host [14,15,16]. Because *C. parapsilosis* is responsible for one-third of neonatal *Candida* infections, with a mortality rate of approximately 10%, low-birth-weight neonates are at especially high risk [17].

The distribution of *C. parapsilosis* recovered from patients with bloodstream infections in various studies conducted in different geographical areas shows that its relative dominance differs according to region [5]. It is the second most common *Candida* isolate in Latin America countries, such as Argentina, Peru and Brazil. In Venezuela and Colombia, *C. parapsilosis* even outranks *C. albicans* infections [5,18,19]. The incidence of *C. parapsilosis* infections in Europe is region-dependent; in Southern European hospitals (Portugal, Spain, Italy, and Greece) it is the second most isolated species [20,21,22,23], and in central and northern countries of Europe the incidence of *C. parapsilosis* ranks third, after that of *C. albicans* and *C. glabrata* [24,25,26]. A different prevalence was also reported in North American countries, Canada and USA, where *C. parapsilosis* ranks second and third, respectively [27,28,29,30]. According to studies of bloodstream fungal infections in Asia (China and Japan), *C. parapsilosis* is commonly found after *C. albicans* [31,32], while in India it ranks third [33]. A similar incidence of infection was observed in Australia [34].

The two cryptic *psilosis* species, *Candida orthopsilosis* and *Candida metapsilosis*, are also opportunistic pathogens, associated with local and systemic diseases. As with *C. parapsilosis*, their frequency and distribution reportedly differ in distinct geographical areas [35,36].

*C. parapsilosis* is a diploid pathogen, with eight chromosome pairs and an estimated genome size of 13.1 Mb. From the 5837 ORFs identified in this species, only 107 (1.83%) have actually been characterized [37]. Its genome is highly conserved; compared to other *Candida* spp., it exhibits a remarkably low level of heterozygosity with just one single nucleotide polymorphism (SNP) per 15,553 bases, more than 70 times less than the corresponding number in the closely related *Lodderomyces elongisporus* [38].

The yeast cells of *C. parapsilosis* display an oval, round, or cylindrical shape, and their colony phenotypes have been identified as crepe, concentric, smooth, or crater [13,39]. Unlike *C. albicans*, *C. parapsilosis* does not form true hyphae; it only exists as yeast or in pseudohyphal forms. Form and colony phenotypes are intimately linked; cells exhibiting crepe and concentric phenotypes are almost entirely pseudohyphal, whereas those with smooth and crater phenotypes are mostly yeast-like [39].

## 3. Virulence Attributes

Similarly to other microorganisms, *Candida* species have developed several specific and effective strategies to enhance their pathogenicity. The virulence of *C. parapsilosis* is mainly attributed to its intrinsic ability to adhere to the abiotic surfaces of medical devices and prosthetic materials, and to the host’s mucosal epithelium. This ability is crucial for biofilm formation and consequently damage to the host [15,40]. 

Researchers have found that the ability to colonize upon mucosal surfaces or inert materials varies among *Candida* species [41]. An unusually high intraspecies variation in terms of adhesion ability has also been identified among clinical isolates of *C. parapsilosis*, compared with other *Candida* species. A correlation between the site of isolation and the rate of adhesion has also been reported, as *C. parapsilosis* mucocutaneous isolates demonstrate higher adhesiveness [41]. 

### 3.1. Cell Adhesion

Adhesion is an important, multifactorial process that is mediated by the characteristics of fungal and host (biotic or abiotic) cells, including cell surface hydrophobicity, cell wall composition, and growth conditions [42]. Initially, the adhesion of the yeast cells is highly dependent upon hydrophobic interactions between the microorganism and host surfaces. Cell surface hydrophobicity is strongly correlated with adhesion to both polystyrene/polyetherurethane surfaces and to epithelial cells. *Candida* species generally exhibit a high degree of cell surface hydrophobicity [43].

In adhesion, the key trigger interaction is promoted by specific cell wall proteins, namely adhesins. This process promotes the attachment of the fungal cells to other microorganisms, the host’s epithelium, and abiotic surfaces [40]. Among *Candida* spp., several adhesin families are involved in adherence. Important adhesin families include: (i) the hyphal wall protein (Hwp) family, which includes five proteins, namely, Hwp1, Hwp2, Rbt1, Eap1, and Ywp1, that play a role in *C. albicans* biofilm formation [42,44]; (ii) the adhesins of the *EPA* (epithelial adhesion) family in *C. glabrata*, comprising 23 genes, of which *EPA1*, *EPA6*, and *EPA7* are described as the most important for the adhesion process in this species [42,44,45]; and (iii) the Als-like (agglutinin-like sequence) family encoding large-cell-surface glycoproteins involved in *Candida* adhesion, including *C. albicans*, *C. parapsilosis*, *C. tropicalis*, *C. dubliniensis*, *C. lusitaniae*, and *C. guilliermondii* [42,44]. Among the eight Als members described in *C. albicans*, Als3 has the most profound impact on biofilm formation; its deletion causes a severe biofilm formation defect [46]. In *C. parapsilosis*, five Als proteins are present on the surface of the pseudohyphae, and the ortholog CaAls7 has been described as a determinant for adhesion to host epithelial cells [47,48]. Other adhesion proteins and non-protein factors with similar properties, such as Eap1, Iff4, Mp65, Ecm33, Utr2, Int1, and Mnt1, have also been identified in *Candida* species; however, these have not been widely studied to date [49].

### 3.2. Secretion of Hydrolytic Enzymes

*Candida* species can produce and secrete several hydrolytic enzymes, including secreted aspartyl proteases (SAPs), lipases (LIPs), and phospholipases. The activity of these enzymes is closely linked with *Candida*’s pathogenicity, such adhesion, cell damage, and the invasion of host tissues [40].

The production of SAPs by *Candida* cells aims to degrade structural and immunological defense proteins in the host, facilitating the invasion and colonization of the host tissue. Compared to *C. albicans*, *C. parapsilosis* expresses less SAP activity [50]. To date, three aspartyl protease-encoding genes (*SAPP1* to *SAPP3*) have been identified in *C. parapsilosis*, with a wide variability in expression among different isolates [51]. Isolates from body surfaces, such as skin or vaginal mucosa, are more invasive than those recovered from systemic infections or from environmental surfaces, due to the production of such enzymes [52].

In addition to SAPs, enzymes categorized as lipases catalyze both the hydrolysis and synthesis of triacylglycerols. Of the four secreted-lipase-encoding genes identified in the *C. parapsilosis* genome, only two (*LIP1* and *LIP2*) have been confirmed as able to encode functionally active proteins. Although the production of LIPs varies greatly among *C. parapsilosis* isolates, ranging from 36% to 80%, their role in enhanced pathogenicity has been confirmed [53]. The putative roles played by LIPs in a successful host invasion include the digestion of lipids for nutrient acquisition, the enhancement of adhesion and biofilm formation, and the suppression of immune response, among others [54,55].

Other hydrolytic enzymes have also been described, including secreted phospholipases, which hydrolyze phospholipids and fatty acids, thereby exposing host receptors and facilitating adhesion; however, these are still poorly understood in *C. parapsilosis* [56].

### 3.3. Biofilm Formation

**Figure 1:**
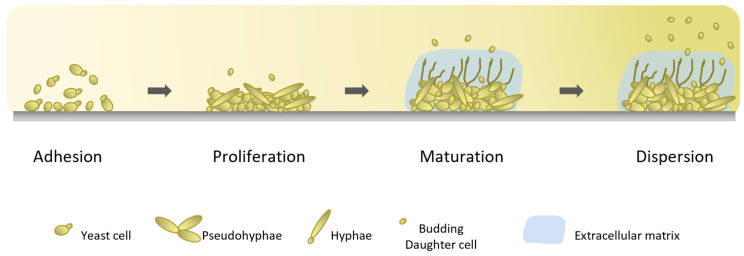
Illustration of biofilm formation cycle in *Candida* spp. Biofilm development consists of three stages: an early phase, in which cells adhere to biotic or abiotic surfaces; an intermediate phase, involving cell proliferation and the formation of a mixed population; and, finally, a maturation/dispersion phase, characterized by the production of the extracellular matrix and the massive dispersion of cells. The detachment and dispersion of daughter cells occurs in all stages of biofilm development.

Biofilms have been described as an organized community, comprising a dense network of microbial cells embedded in an extracellular matrix (ECM) of polymers [13]. Biofilm formation is a potent virulence attribute of several *Candida* species. Biofilm formation during infection has been linked to higher mortality rates in cases involving such species when compared with isolates incapable of forming biofilm [57]. Biofilm development is a well-regulated process comprising three sequential stages (Figure 1): an early phase, involving the entire adhesion process of the cells, as described above; an intermediated phase, and, finally, a maturation/dispersion phase [40]. In the intermediate phase, following initial fungal adhesion, yeast cells undergo a morphology transition from yeast to filamentous or pseudohyphal forms, forming a mixed population with a multilayer formation (Figure 1). Afterwards, biofilm maturation begins through the production and secretion of a polysaccharide-rich extracellular matrix, formed by polysaccharides, proteins, lipids, and nucleic acids, which provides structural and functional stability to the biofilm [40,58]. 

The biofilm’s architecture, morphology, and thickness also vary widely among *Candida* species and between strains [58]. These features are influenced by several host and *Candida*-derived variables, including: (i) physiological conditions, such as pH and oxygen concentration; (ii) fluid flow at the infection site, which influences nutrient exchange and impacts the biofilm’s structural integrity; (iii) available nutrients in the growth media, including sugars, lipids, and serum; and (iv) the material on which the biofilm grows (those typically used in medical devices include silicone, latex, and polyurethane, among others); and (v) community microbial interactions, either fungal–fungal or fungal–bacterial, which modulate the ability of *Candida* to form biofilm and also represent a promising topic for future research [58,59,60].

*C. parapsilosis* biofilm growth is especially common in patients fitted with a central venous catheter who receive total parenteral nutrition [61,62]. The biofilm structure of *C. parapsilosis* exhibits high variability among clinical isolates. Because *C. parapsilosis* does not form true hyphae, its biofilm is composed of aggregated blastoconidia and pseudohyphae that occupy a volume lower than that of other *Candida* species [63,64]. In addition, the extracellular matrix of *C. parapsilosis* biofilm is mainly composed of carbohydrates and low levels of protein [63].

The ability to form biofilms is closely related to its virulence potential, because only limited penetration of substances is possible through the biofilm matrix, resulting in a greatly decreased susceptibility to antimicrobial agents [65,66]. The development of the biofilm also serves to counter the host immune response by inhibiting macrophage phagocytosis and antibody activity [65]. 

The process of biofilm development involves a massive cell detachment during the final maturation phase, with consequent dispersion that promotes the colonization of new locations and surfaces [40]. However, Uppuluri et al. [67] found that dispersion was not confined to the maturation phase and occurs continuously during the biofilm development process. A more robust biofilm is produced by dispersed cells compared with the biofilm formed by initial planktonic mother cells such that the virulence potential increases over generations. All of these findings represent matters of serious clinical concern, not only for the treatment of patient infections but also in terms of public health [66].

The complexity of all stages of biofilm formation, involving such phenomena as the control of adhesion, morphology changes, and ECM production, among others, requires an extensive and complex regulatory network [68]. The biofilm formation regulatory process has been extensively studied in *C. albicans*; however, as with other characteristics, such knowledge cannot be simply transposed to other *Candida* species. For example, the four transcription factors *BRG1*, *TEC1*, *ROB1*, and *FLO8* are all involved in the biofilm regulatory network of *C. albicans* but play no role in the biofilm regulation of *C. parapsilosis* [68,69]. Conversely, *CZF1*, *UME6*, *GZF3*, and *CPH2* have been highlighted as key contributors to biofilm formation in *C. parapsilosis*, but these genes play a negligible role in this process in *C. albicans*. One recent report identified the direct role of Ndt80 as a repressor of *C. parapsilosis* virulence attributes, thereby diverging functionally from its homolog in the closely related fungal pathogen *C. albicans* [70]. However, other genes required for biofilm development, such as *ACE2*, *BCR1*, and *EFG1*, have been found to perform a similar function in both species [68,71].

## 4. Antifungals and Resistance Mechanisms

Despite ongoing research efforts concerning new therapeutic compounds and treatment strategies, only a limited number of options of antifungal drugs are available for the treatment of candidosis [72]. Currently, the arsenal of systemic antifungals available for clinical use consists of only three major drug classes: polyenes, echinocandins, and azoles [73].

### 4.1. Polyenes

**Figure 2:**
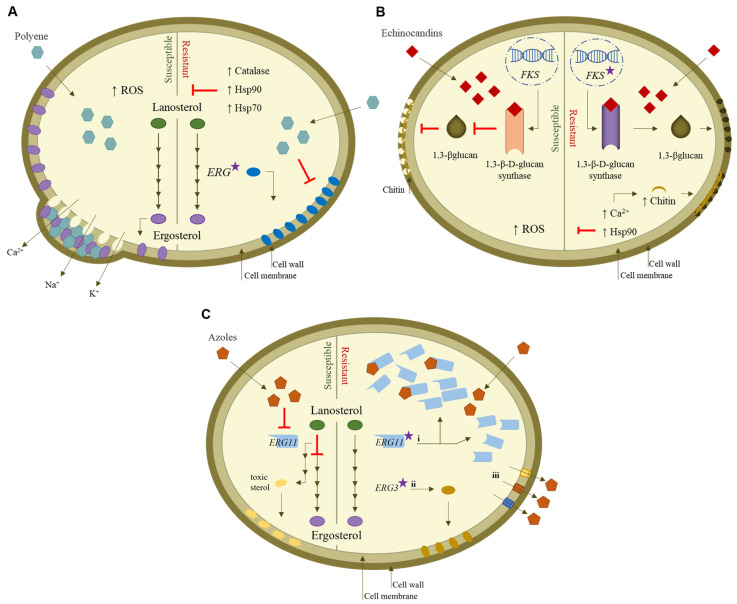
Mechanism of action of antifungals against *Candida* spp. and mechanisms underlying drug resistance. (**A**) Polyenes act by forming polyene/ergosterol aggregates, destabilizing the fungal membrane. The action of polyenes can be overcome through mutations in ergosterol biosynthesis genes responsible for altered sterol composition and by the activation of stress response pathways, such as catalase and Hsp. (**B**) Echinocandins act as noncompetitive inhibitors of (1,3)-β-D-glucan synthase, encoded by *FKS* genes, causing a depletion of the 1,3-β-glucan in the cell wall. Echinocandin resistance in *Candida* is associated with mutations in *FKS* genes and the activation of cell wall stress response mediator pathways, such as Hsp90 and calcineurin (Ca^2+^), increasing the chitin content. (**C**) Azoles target and inhibit the enzyme lanosterol 14α-demethylase, encoded by the *ERG11* gene, leading to the accumulation of toxic sterol. Azole resistance involves: (i) point mutations in the *ERG11* gene, which can be responsible for its overexpression and/or the inhibition of enzyme lanosterol 14α-demethylase, due to the decrease in azole–target binding affinity; (ii) mutations in *ERG* genes involved in the ergosterol biosynthesis pathway, particularly in *ERG3*; and (iii) increased efflux of the azole drugs from the fungal cell through the overexpression of multidrug efflux pumps. Red T-shaped bars indicate inhibition. Star icon indicates gene mutation.

Amphotericin B (AmB) is the most used member of the class of polyenes, being clinically used for more than 55 years [73]. Its potent fungicidal activity is derived from its interaction with the ergosterol of fungal cells by binding to the lipid bilayer, forming pores in the cell membrane and facilitating the leakage of intracellular components, such as potassium ions (K^+^), into the extracellular medium (Figure 2A) [74]. Consequently, this interaction results in a drastic change in cell permeability, ultimately leading to cell lysis. This antifungal has low solubility and is highly toxic to the host cell due to the close structural relationship between ergosterol and cholesterol, the mammalian membrane sterol. This limits its use in long-term antifungal therapy [75]. However, less toxic, lipid-based polyene formulations have now been developed, including liposomal amphotericin B (LAmB), which has become the first-line treatment for various types of invasive fungal infections [76].

The development of fungal resistance to polyenes is rare. Most *Candida* spp., including *C. albicans*, *C. glabrata*, and *C. parapsilosis*, are generally considered to be susceptible to AmB, with surveillance studies reporting an AmB susceptibility rate close to 100% [77]. Recently, a global pooled prevalence meta-analysis estimated *C. parapsilosis* AmB-resistance at 1.3% [78]. Emerging AmB resistance has been reported in species, such as *C. auris* [79]. The resistance mechanisms of this class are less well understood than those of echinocandins and azoles; nevertheless, several hypotheses have been forwarded to explain resistance, as illustrated in Figure 2A. These include: (i) sterol composition modulation through the depletion or replacement of ergosterol triggered by mutations in genes involved in the ergosterol biosynthesis pathway, specifically in *ERG1* to *ERG4*, *ERG6*, and *ERG11* [80,81,82]; (ii) enhanced defense against oxidative damage to break down the reactive oxygen species (ROS) that are produced under AmB exposure, either by means of catalase activity and/or by the molecular chaperones of the heat shock protein (HSP) family, namely, Hsp90 and Hsp70 [83,84,85].

### 4.2. Echinocandins

Echinocandins, i.e., caspofungin, micafungin, and anidulafungin, are the newest class of antifungal drugs available for the treatment of invasive fungal infections and offer an excellent safety profile combined with high fungicidal activity [86,87]. They noncompetitively inhibit (1,3)-β-D-glucan synthase, which is responsible for the biosynthesis of 1,3-β-D-glucan, a crucial structural component of fungal cell walls [88,89]. Specifically, echinocandins target the catalytic subunits *FKS1* of β-D-glucan synthase, encoded by *FKS1* and *FKS2* genes, leading to the disruption of cell wall glucan, osmotic instability, cell lysis, and death for most species (Figure 2B) [90,91]. Although their antifungal spectrum is limited, echinocandins are fungicidal against most *Candida* spp., including azole-resistant strains and biofilm [92,93]. However, as the use of these drugs has expanded, reports of resistance to echinocandin treatments among *Candida* spp. have increased [93]. In particular, *C. parapsilosis* tends to be associated with increased in vitro minimum inhibitory concentrations (MICs) of echinocandin [94,95], raising concerns that such drugs may facilitated the development of high levels of resistance [96,97,98].

Decreased echinocandin susceptibility can occur via two main mechanisms (Figure 2B): (i) an adaptive stress response mechanism, involving a compensatory increase in the synthesis of chitin (an essential cell wall component) that is mediated, for example, via the activation of the calcineurin (Ca^2+^) signaling pathway. The activation of this pathway is initially signaled by the Hsp90 chaperone, a key regulator of cellular stress response, and thus confers protection against the antifungal agent [99,100,101]; (ii) acquired or intrinsic mutations in genes encoding *FKS1* and *FKS2*, characterized by amino acid substitutions in specific regions clustered around two highly conserved regions (termed hot spots 1 and 2) of *Fksp*, which is generally correlated with increased resistance to such drugs [95,102,103]. Acquired mutations have been reported for *C. albicans*, *C. tropicalis*, *C. krusei*, and *C. glabrata* [102,104] but not yet for *C. parapsilosis* [96,105]. In *C. parapsilosis,* naturally occurring *FKS1* mutations in the hot spot 1 region were found to be responsible for the intrinsic reduced susceptibility of this species to echinocandins [106]. 

### 4.3. Azoles

Azoles represent the largest class of antifungal agents in clinical use due to their broad spectrum of activity, favorable safety profile, and bioavailability [73]. The clinically approved azoles include fluconazole (FLC), voriconazole (VRC), posaconazole (PSC), itraconazole, and isavuconazole. Azoles exhibit mainly fungistatic activity against *Candida* [107]. Due to differences between the membranes of fungal and human cells (mainly composed of cholesterol), the use of azoles does not interfere with human body cells during treatment. They bind to and inhibit the activity of the enzyme lanosterol 14α-demethylase (encoded by the *ERG11* gene in yeasts), which is a key enzyme in the ergosterol biosynthetic pathway (Figure 2C) [108,109,110]. Ergosterol is an important component of fungal cell membranes [111]. The interruption of its synthesis enables the accumulation of a toxic 14α-methyl sterol, which impairs the membrane integrity and also the function of some membrane-bound proteins (such as those involved in cell wall synthesis), with consequences in terms of cell growth [108,111,112]. 

The emergence of azole resistance in *Candida* species represents a major challenge to treatment [113,114,115,116]. *Candida* spp. azole resistance has been linked to different molecular mechanisms that include (Figure 2C): (i) mutations in the gene encoding the azole target enzyme lanosterol 14α-demethylase (*ERG11*), with resulting overexpression, and reduced azole binding, which also results in the reduction in or loss of affinity with azoles, preventing azole binding; (ii) alterations in the ergosterol biosynthetic pathway, caused by loss-of-function point mutations in *ERG3*, leading to a depletion of ergosterol and to the accumulation of 14α-methyl fecosterol, which is less damaging to cell membranes, thus enabling continued growth in the presence of azoles; and (iii) the upregulation of multidrug efflux pumps *CDR1* and *CDR2* (*Candida* drug resistance) and *MDR1* (multidrug resistance) genes that transport the drug out of the cells [117,118]. The analysis of serial isolates from individual patients has revealed that acquired azole resistance commonly relies on multiple and often-combined molecular mechanisms [119].

Similarly to *C. albicans*, *C. parapsilosis* harbors several genes that have been found to be involved in resistance development. For example, Mrr1p (multidrug resistance regulator 1) is a zinc cluster transcription factor that controls *MDR1* expression [120]. Several authors have demonstrated that gain-of-function mutations in the *MRR1* gene, which render the transcription factor constitutively active, are responsible for the upregulation of the *MDR1* efflux pump and thus play a central role in the development of drug resistance [121,122,123,124]. The hyperactivation of the Tac1 (transcriptional activator of *CDR* genes 1) transcription factor is also conferred by gain-of-function mutations that consequently promote the overexpression of *CDR1* and *CDR2* genes [125,126]. Recently, researchers described a new azole resistance mechanism in *Candida*, particularly among *C. parapsilosis* isolates, involving another Cdr1-like gene, the *CDR1B* (CPAR2_304370). Expression of a GOF mutation in the *MRR1* gene impacts the fluconazole susceptibility in *C. parapsilosis* through *CDR1B* overexpression [114,127]. *CDR1* (CLUG_03113) expression in *Candida lusitaniae* is also shown to be regulated by GOF mutation in *MRR1* [128]. In addition, several pieces of evidence point to another mechanism involved in *C. parapsilosis* antifungal resistance: allele copy number variation. Our group observed an increase in the *CDR1B* copy number, resulting in *CDR1B* overexpression and a consequent reduction in fluconazole susceptibility [114]. The copy number variation mechanism has not only been associated with the drug fluconazole but also with miltefosine, a drug recently approved by the FDA for the treatment of invasive candidiasis [129].

Upc2 (Sterol uptake control protein 2), another member of the zinc cluster transcription factor family, is a key regulator of ergosterol metabolism that controls the expression of the azole target *ERG11* gene [130,131,132]. Gain-of-function mutations in *UPC2* lead to the increased *ERG11* expression, contributing to fluconazole resistance in this species [133,134,135]. As with *UPC2*, the transcription factor Ndt80 also modulates the expression of several ergosterol metabolism genes [132,136]. Moreover, Chen et al. (2004) demonstrated the involvement of this regulatory factor in azole tolerance by controlling the expression of the *CDR1* gene in *C. albicans* [137].

Alterations in the ergosterol biosynthetic pathway, including mutations in the *ERG11* gene or its overexpression, have also been linked to azole resistance [138]. The amino acid Y132F substitution in *ERG11* is frequently reported among *Candida* spp., including *C. parapsilosis* [113,139,140,141,142]. The persistence of *C. parapsilosis* isolates harboring the Y132F mutation in clinical settings has been associated with outbreaks of infections in hospitals, with fatal consequences [115,116,143].

## 5. Final Remarks

*Candida parapsilosis* is a predominant species within NACs that is responsible for invasive candidosis in low-birth-weight neonates, transplant recipients, critical care patients, and those receiving parenteral nutrition. The high prevalence of *C. parapsilosis* is also promoted by its well-documented ability to persist and thrive in the hospital environments for long periods. Its remarkable ability to adhere to abiotic surfaces, such as catheters, and to form biofilms constitutes a gateway to systemic colonization. The extensive use of antifungals, both prophylactically and therapeutically, is also recognized as a major cause of worldwide antifungal resistance in this pathogen.

In light of the above, there can be no doubt that further comprehensive research efforts addressing the epidemiology, pathogenic attributes, antimicrobial susceptibility profile, and genetic resistance mechanisms of *Candida parapsilosis* will contribute to improved treatments for and the prevention of infections, leading to improved patient outcomes and lower burdens upon healthcare systems.

## Data Availability

All data are publicly available.
